# Supplementary material for: A concentric tube catheter for endoluminal interventions, steered and imaged via magnetic resonance imaging
Source: Commun Eng. 2026 Mar 9;5:74. doi: 10.1038/s44172-026-00636-1 (PMC13100120; doi:10.1038/s44172-026-00636-1)
Supplement: Supplementary file 2 — Supplemental Material [file 44172_2026_636_MOESM2_ESM.pdf]

Supplementary Material - A Concentric Tube Catheter for Endoluminal Interventions, Steered and Imaged via Magnetic Resonance Imaging, Lloyd and Murasovs

Supplementary Methods

S1 The Rigid Link Model

The system is modelled as a chain of pseudo-rigid links (Fig. S1) as described in detail in[1]. The rigid link model encapsulates bending and twisting behaviour but, in common with the Kirchhoff rod model, assumes shear stress and extension/compression to be zero. The continuum structure is discretized into n rigid links, each with a 3D joint, with the joint angle array given as:

$$\mathbf{Q} \in \mathbb{R}^{3n\times 1} = \begin{bmatrix}\mathbf{q}_0 & \cdots & \mathbf{q}_i & \cdots & \mathbf{q}_n\end{bmatrix}^T . \tag{S1}$$

Where $\boldsymbol{q}_i$ represents the 3D joint angle at the i'th link. Joint stiffness is determined by the linear stiffness matrix (Supplementary Method S2):

$$\mathbf{K} \in \mathbb{R}^{3n\times 3n} = \underbrace{diag(E_xI_x, E_yI_y, GJ)/L}_{\text{repeated diagonally } n \text{ times}} . \tag{S2}$$

Where E_xI_x and E_yI_y comprise the elastic modulus and second moment of area and constitute bending stiffness about X and Y respectively and GJ comprise the shear modulus and the polar moment of area and constitute torsional stiffness about Z. L is the length of each joint. For extreme deformations (strain $>$ 100%), the assumptions of linear elasticity (Equation S2) break down but for the lower, linear strains we experience in this work, a relatively small number of links ($n \approx 16$) can be shown to be mesh independent.

The magnetic torque $\boldsymbol{\tau}_{mag}$ of each link is stacked into a wrench vector as:

$$\mathbf{S} \in \mathbb{R}^{6n\times 1} = \begin{bmatrix}0_{3\times 1} & \boldsymbol{\tau}_{mag_0} & \cdots & 0_{3\times 1} & \boldsymbol{\tau}_{mag_i} & \cdots & 0_{3\times 1} & \boldsymbol{\tau}_{mag_n}\end{bmatrix}^T \tag{S3}$$

with the $0_{3\times 1}$ components representing the contribution of magnetic force, zero in our homogeneous background field. This torque is applied at the locations of the magnetic rings (or pin, in the case of sleeve A) as described in Supplementary Method S3 and set to zero for the magnetically inert sections of the CoSMA.

Finally, a complete expression balancing magnetic and elastic torques can be written as:

$$\mathbf{K}\mathbf{Q} = \mathbf{J}^T\mathbf{S} \tag{S4}$$

where $\mathbf{J}^T \in \mathbb{R}^{3n\times 6n}$ is the transpose of the differential kinematic Jacobian, itself a function of $\mathbf{Q}$ [1]. Consequently, no closed form solution exists and a convergent numerical solution must be applied as described in Methods.

S2 Mechanical Characterization

In order to determine the mechanical properties of the two braids, an experiment was devised employing remnant magnetic torque in a low field ($<$ 10 mT) Helmholtz coil. Results are processed

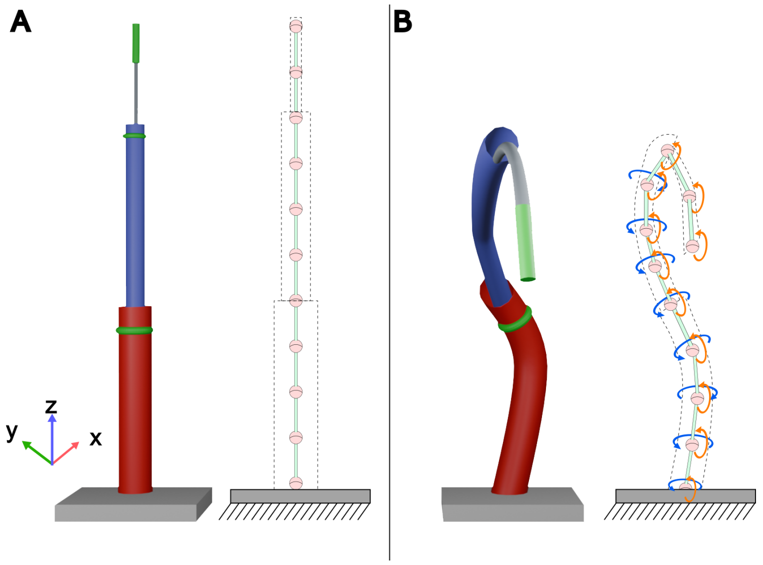

Figure S1: The 3D pseudo-rigid link model. (**A**) in referential pose and (**B**) in deformed pose. Magnetic torques are imparted at the locations of the magnetic elements (shown in green). These are balanced against elastic torques at each pseudo-joint. The full shape of the structure is encapsulated by the joint angle array ($\boldsymbol{Q}$) from which a Jacobian transpose ($\boldsymbol{J}^T$) can be constructed to map magnetic wrench ($\boldsymbol{S}$) to joint stiffness ($\boldsymbol{K}$). As detailed in Methods, the three sleeves are independently modelled and then connected by interactive torques.

based on the modeling presented in Supplementary MethodS1. Any permanent magnet of magnetization $\boldsymbol{m}$ will experience an aligning torque in a background field ($\boldsymbol{\tau} = \boldsymbol{m} \times \boldsymbol{B}$) [2]. This known torque can thus be exploited to determine the elastic restoring forces within an anisotropic material such as our braid. By independently arranging the cross product about the Y and Z axis (see Fig. S1 for axes definitions) respectively, the stiffness of the braid can be determined under the isolated bending and twisting primitives.

S2.1 Bending Deformation

Each braid had a permanent magnet (4 mm length X 3 mm diameter, N52) attached to the distal end with magnetization aligned along the y-axis and was constrained at 20 mm and 30 mm lengths in the centre of our 3-D Helmholtz coil (3DXHC12.5-300, Dexing Magnet Tech. Co., Ltd, Xiamen, China) as shown in Fig. S2A. A homogeneous magnetic field was swept from -10 mT to +10 mT in the z-direction (-8 mT to +8 mT for sleeve B to avoid extreme deformation) and the results recorded via a Basler 25 mm zoom video camera (docs.baslerweb.com). Apple Keynote was used to gain the deformed tip angle from the still frames at each field. Given a remnant magnetization of 1.43 T, known magnet dimensions, magnet density of 7500 kg/m^3 for the gravity vector - added as a force to Equation S3, and known applied fields, Equation S4 can be solved to determine the bending stiffness (EI) of the braids. The bending stiffness of the 3 mm diameter braid (sleeve C) was calculated as $1.6 \times 10^{-6} \pm 2.1 \times 10^{-7} Nm^2$ and that of the 1.5 mm braid (sleeve B) as $1.0 \times 10^{-6} \pm 1.3 \times 10^{-7} Nm^2$. Sleeve A is manufactured from 0.4 mm diameter isotropic nylon (elastic modulus $E \approx 1\ GPa$, https://www.plastics.toray/technical) giving a calculated bending stiffness of EI $= 1.3 \times 10^{-6} Nm^2$. For the sake of comparison, a bending stiffness of $1.6 \times 10^{-6} Nm^2$ is equivalent to a Dragon Skin-10 catheter (elastic modulus $E \approx 200\ kPa$ [3]) of diameter 3.6 mm. Fig. S3 illustrates these bending stiffnesses in comparison with Nitinol concentric tube robots and clinical manual guidewires, catheters and sheaths.

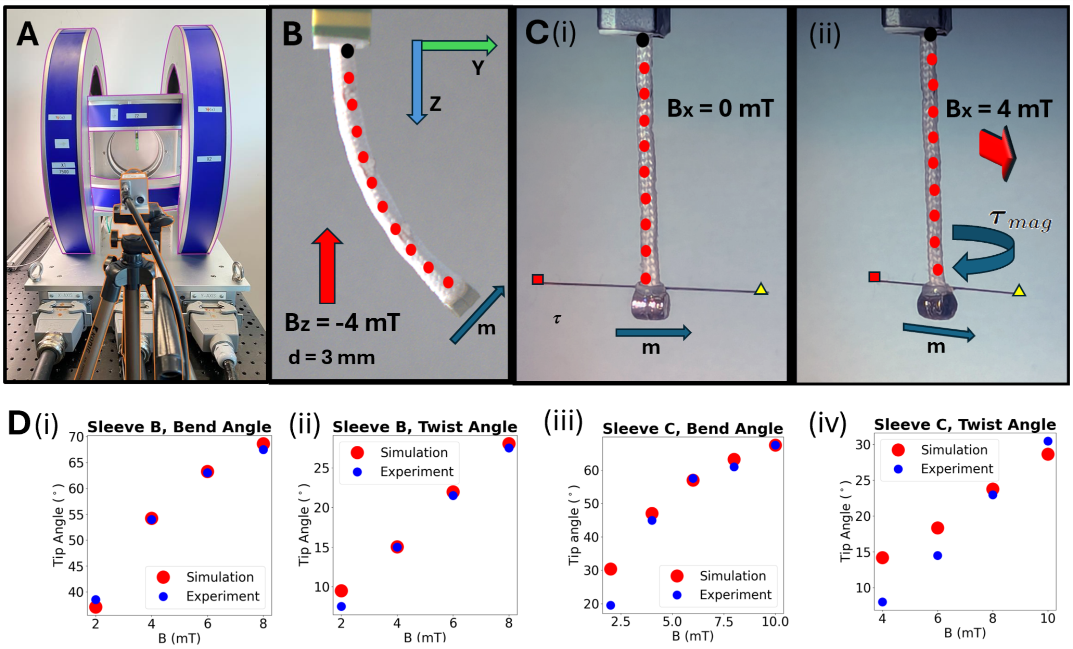

Figure S2: (A) The deformation experiment setup in the 3D Helmholtz coil (pink). The braid with magnet attached (green) is constrained with a 3D printed clip (blue) and the data gathered via a Basler camera (orange). (B) The 3 mm diameter braid (Sleeve C) subjected to a pure bending torque induced by a -4 mT field in our 3D Helmholtz coil with the results of the rigid link simulation projected in red. (C) The corresponding pure twisting torque on the same 3 mm diameter braid (Sleeve C). A Nitinol bar is attached to the magnet to extract pose data at (i) B = 0 and (ii) B = 4 mT in this example. (D) Graph of actuating field against tip angle for simulation (red) and experiment (blue). (i) The 1.5 mm diameter braid (sleeve B) under the bending primitive. (ii) The 1.5 mm diameter braid (sleeve B) under the twisting primitive. (iii) The 3 mm diameter braid (sleeve B) under the bending primitive. (iv) The 3 mm diameter braid (sleeve C) under the twisting primitive.

Table S1: Comparison of Elastic (Young's) modulus of various catheter designs against various anatomical tissues. The present contribution is still stiffer than the more sensitive anatomical regions but represents a significant improvement on the state of the art.

**Material/Design**	**Elastic Modulus (Nm^{-2})**
This work, Sleeves B and C	$500kPa$
This work, Sleeve A	$1 \times 10^3 kPa$
Elastic Concentric Tube Robots	$50 \times 10^6 kPa$
Manual Sheaths/Guidewires	$8 \times 10^3 - 500 \times 10^3 kPa$
Arterial Tissue [7]	$160 - 360kPa$
Lung Tissue [8]	$2.5 - 4kPa$
Brain Tissue [9]	$0.5 - 1kPa$

S2.2 Twisting Deformation

Using an identical setup as the deformation experiment with the exception of applied field orientation (See Fig. S2C), each braid was constrained again at 20 mm and 30 mm lengths. A magnetic field in the z-direction was applied to induce torque in the braid. Results were again analyzed using Apple Keynote and Equation S4 solved to determine the twisting stiffness (GJ) of the respective braids. In this arrangement the influence of gravity is zero. The twisting stiffness of the 3 mm diameter braid

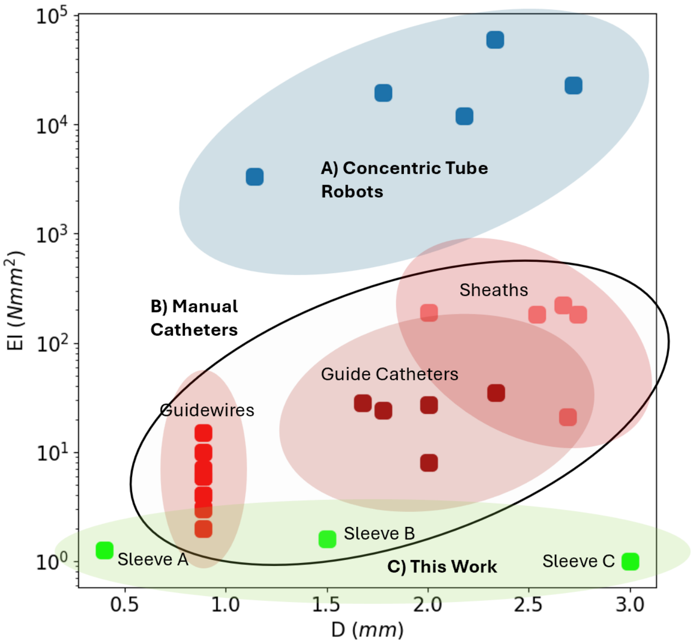

Figure S3: Comparison of Bending Stiffness (EI) against Diameter (D) for the state-of-the-art in: (A) Super-elastic concentric tube robots (in blue, taken from [4, 5]. (B) Clinical manual guidewires, catheters and sheaths (in red, taken from [6]). (C) This work (in green, taken from Supplementary Method S2).

(sleeve C) was calculated as $1.9 \times 10^{-5} \pm 4.0 \times 10^{-6} Nm^2$ and that of the 1.5 mm braid (sleeve B) as $1.4 \times 10^{-5} \pm 2.1 \times 10^{-6} Nm^2$.

According to linear elasticity theory, for a slender rod of isotropic material, $\frac{GJ}{EI} = \frac{2}{3}$. For our 3 mm diameter braid (sleeve C) $\frac{GJ}{EI} = 12$. For our 1.5 mm diameter braid (sleeve B) $\frac{GJ}{EI} = 14$. This illustrates the $\approx 20X$ increase in ratio of twisting to bending stiffness afforded by the anisotropic behaviour of the braid, similar to that reported in [10]. This ratio $\frac{GJ}{EI}$ is fundamentally important to the snap-through instability exhibited in conventional concentric tube robots [11]. Using our proposed actuation strategy it becomes possible to exploit materials with both a much lower overall stiffness as well as a lower EI with respect to GJ. This is enabled as the magnetically induced torques (as compared to mechanically induced) significantly reduce transmission stiffness requirements.

S3 Magnetic Characterization

Ferromagnetism is the property of a (not necessarily, but often, iron-based) material to display a strongly positive material susceptibility ($\chi \gg 0$) [12]. That is, to induce a parallel magnetic moment when subject to an applied field. Other forms of magnetically responsive material do exist (dia-, para-, ferri- and antiferro-magnetic), but, for the purposes of robotic actuation, display too weak a response to be considered here [12]. In order to determine the torque on a non-spherical ferromagnetic body in a background field we must first review the concept of demagnetization.

A ferromagnetic object exposed to a background field ($\mathbf{H_b}$) generates a demagnetizing field ($\mathbf{H_d}$) which acts to reduce the overall magnetic moment. The total field at any point is the sum of these two fields ($\mathbf{H} = \mathbf{H_b} + \mathbf{H_d}$) and the induced magnetization is related to the total field via the apparent susceptibility tensor, $\mathbf{M} = \boldsymbol{\chi_a}.\mathbf{H}$ [13] (Here the upper-case $\mathbf{M}$ denotes magnetization per unit volume in A/m, the same units as the field $\mathbf{H}$ thus $\boldsymbol{\chi_a}$ is dimensionless). The apparent susceptiblity tensor ($\boldsymbol{\chi_a} \in \mathbb{R}^{3\times3}$) can be shown, for sufficiently high material susceptibility ($\chi > 10^3$) to be inversely proportional to the demagnetization factors along the principle axes of the body

$\boldsymbol{\chi_a} = diag(1/n_x, 1/n_y, 1/n_z)$ which, counter-intuitively, are purely a function of geometry [2]. In a strong background field, these demagnetization factors allow for exact determination of the magneto-static energy. Demagnetization factors are subject to the constraint $n_x + n_y + n_z = 1$ (for finite rings [14]) and, for radially symmetrical geometries, can be reduced to 1D as $n_y == n_z$. For the sake of convention, we rename the demagnetization factor in the axial direction as n_a and the two demagnetization factors in the radial directions as $n_r = (1 - n_a)/2$. Under ultra-high fields, such as our MRI system, saturation magnetization (m_s) will always be attained $|\mathbf{M}| \gg m_s$, thus, magnetic torque can be shown to be as given in Equation 1.

The demagnetizing fields of non-spherical geometries in a background field is an extensive area of physics research [14]. Here we touch on the subject with the express purpose of ascertaining the torque imparted on our magnetically active elements. According to Equation 1, if the demagnetization factors are known, then magnetic torque becomes a function of θ, the angle between the symmetrical axis of the magnetic element and the applied field. Consequently, this section is dedicated to determination of demagnetization factors (Supplementary Method S3.1) and then a reconciliation of the magnetic torques on these shapes when placed in the MRI bore (Supplementary Method S3.2).

S3.1 Magneto-static Finite Element Model

The calculation of demagnetization factors relies on the assumption of uniform magnetization, implying a very strong applied field. This is not typically the case for robotic manipulation or actuation tasks [2], but is true in our case due to the large background field of the MRI system. For shapes which can be approximated to ellipsoids of varying aspect ratio (i.e. a sphere or a rod) analytical solutions exist for these demagnetizing factors [2]. Analytical solutions have been addressed for more complex shapes such as hollow cylinders and shells [14, 15] but characterizing these geometries is cumbersome and such methods always require approximations. For the complex geometry presented here, that of a helical winding, the demagnetization factors must be determined experimentally or numerically (or both) [2].

For the numerical solution, a Finite Element Model (FEM) was constructed in the magneto-statics module of COMSOL multiphysics v6 (COMSOL AB, Stockholm, Sweden). Defining U as the scalar magnetic potential (in A) such that total field $\boldsymbol{H} = -\nabla U$ (in A/m). Because $\boldsymbol{H} = \boldsymbol{B}/\mu_0 - \boldsymbol{m}$ and $\nabla . \boldsymbol{B} == 0$ (where $\boldsymbol{B}$ is the magnetic flux density in T and $\boldsymbol{m}$ is the magnetic moment in A/m), $\nabla^2 U = \nabla \boldsymbol{m}$ within the material domain and $\nabla^2 U = 0$ outside the material domain (where $\boldsymbol{m} = 0$). Numerically solving these second order differential equations over a 500,000 node mesh generates a solution for the point-wise magnetization. This can be volumetrically integrated to determine the bulk magnetization vector ($\boldsymbol{M}$) which, given a known applied field, tells us the demagnetization factors from $\mathbf{M} = \boldsymbol{\chi_a} \boldsymbol{H}$.

The numerical solution will solve for any geometry (as long as it can be robustly meshed) so, as an initial reconciliation of the FEM, we determine axial and radial demagnetization factors for the perfect ellipsoid presented analytically in [13] (Fig. S4 **A**,**B**). An RMS error below 1% confirms the accuracy of the numerical model described above. We then develop the model to represent the geometries of interest as shown in Fig. S4 **C-F** generating the demagnetization factors shown in Table S2. These numbers are inputs to the reconciliation presented in Supplementary Method S3.2.

S3.2 Reconciliation of Magnetic Torques

Utilizing the rigid link simulation detailed in Supplementary Method S2 in conjunction with magnetic properties from Supplementary Method S3.1 we reconciled the deformation of single iron rings of

Table S2: Summary of demagnetization factors obtained via the FEM described in Supplementary Method S3.1 for the geometry shown in Fig. S4 **C**. The wire is annealed iron (99.5% purity, $m_s = 1.43 \times 10^6 A/m$) with a diameter of 0.25 mm. In the upper calculation, the loop has an outer diameter of 4.0 mm and in the lower calculation, an outer diameter of 2.1 mm (See Supplementary Method S6). The magnetic torque (τ_{mag}) is calculated according to Equation 1 at peak ($\theta = 45°$).

	n_a	n_r	volume (mm^3)	$\tau_{mag}(mNm)$ at $\theta = 45°$
Ring Outer Diameter = 4.0 mm	0.95	0.03	0.62	0.73
Ring Outer Diameter = 2.1 mm	0.86	0.07	0.31	0.31

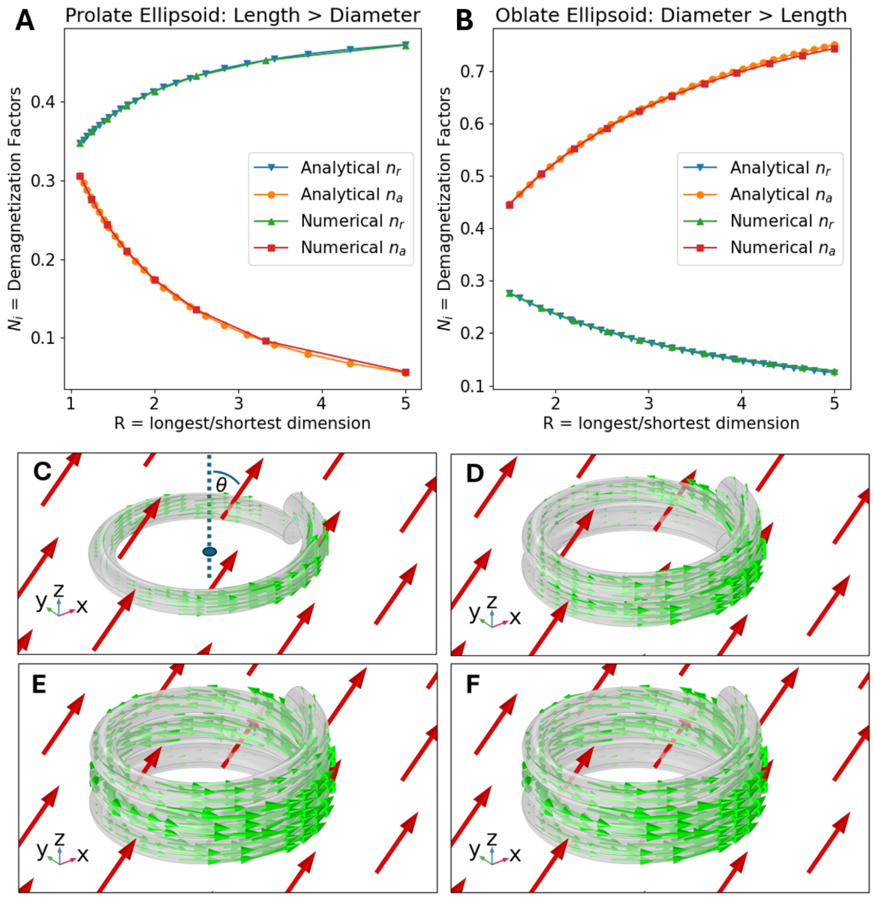

Figure S4: Results from the FEM. Comparison of demagnetization factors for prolate (**A**) and oblate (**B**) ellipsoids determined analytically from the formulae in [13] and via FEM according to the methodology detailed in Supplementary Method S3.1. The RMS error here is below 1% in both scenarios confirming the validity of the FEM. This numerical approach can be thus generalized to complex geometries where comparative analytical solutions do not exist. A 4.0mm diameter iron ring of (**C**) 1, (**D**) 2, (**E**) 3 and (**F**) 4 loops exposed to an arbitrary background field (red arrows). The demagnetizing field is solved over a 500,000 element mesh and the ratio of the volume integral of the induced magnetic moment (green arrows) to the applied field (red arrows) gives the susceptibility tensor ($\boldsymbol{\chi_a}$). This is a function purely of geometry and the results can thus be directly imported into the CoSMA simulation. (**C**) also shows the symmetrical axis of the ring and the angle (θ) between this axis and the applied field which drives Equation 1.

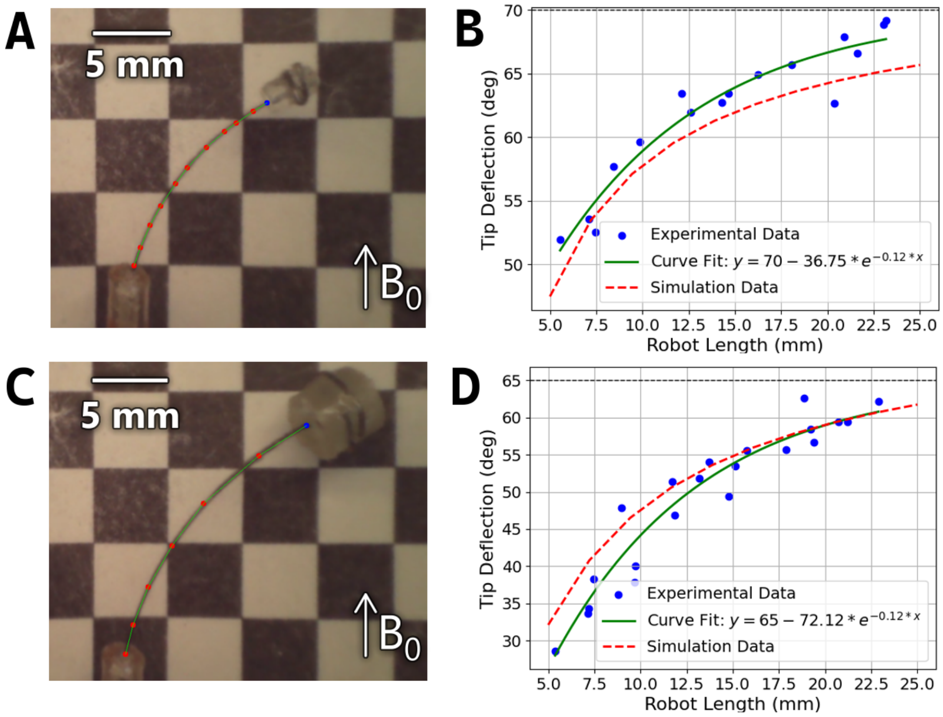

Figure S5: A single ring of iron wire near-orthogonally mounted at the tip of a length of Nitinol. The referential angle between B_0 and the symmetrical axis of the ring (θ_0) must be non-zero (to avoid model singularity) but arises mainly due to manual fabrication inaccuracy. θ_0 is measured for each specimen from the optical images and included as a variable in the rigid link model. (A) A 2.1 mm diameter ring was attached to 0.15 mm diameter Nitinol wire and placed in our 7T MRI bore. The unconstrained length of Nitinol was varied between 5 mm and 25 mm. (B) Three specimens were tested with $\overline{\theta_0} = 20°$. All experimental data-points appear as blue dots, the aggregated curve of these points - tip angle versus manipulator length (green curve) - appears against the corresponding deformations determined via the simulation (red curve) described in Supplementary Method S1. Mean vertical projection percentage error across all data-points for the 2.1 mm diameter test was 4.5%. (C) The process is repeated for the 4.0 mm diameter ring on a 0.20 mm diameter Nitinol wire. (D) Three further specimens were tested with $\overline{\theta_0} = 25°$. Mean vertical projection percentage error across all data-points for the 4.0 mm diameter test was 2.9%.

both 4.0 mm diameter and 2.1 mm diameter over varying lengths ($L \in (5, 25)$ mm) in the bore of the MRI (Fig. S5). The experimental arrangement, harvesting and post-processing of optical images is consistent with the Clinical Applicability Section. For the 4.0 mm diameter specimens, 0.2 mm diameter Nitinol was used as the deforming elastica, for the 2.1 mm diameter specimens, 0.15 mm diameter Nitinol was used. In all cases the Elastic modulus of Nitinol was assumed to be 70 GPa [16]. Each setting was repeated for three identically manufactured specimens. θ_0 - the referential angle between B_0 and the symmetrical axis of the ring was measured for each specimen from the optical images and included as a variable in the rigid link model. Vertical projection error was computed as the difference between the measured tip deflection from the real-world results and the corresponding deflection value predicted by the simulation data at the same manipulator length. From Fig. S5 the deformed tip angle, experimental versus simulation gives, for the 4.0 mm diameter experiment, RMS error of 2.0° (4.5% of mean tip angle) and for the 2.1 mm diameter experiment, RMS error of 1.8° (2.9% of mean tip angle).

S3.3 Variation in Number of Windings

The rings affixed to the sleeves have, in our demonstrations, contained only one loop. This is to minimize the size of the signal void. It is possible to increase the number of loops on each ring (Fig. S4) which increases both torque (Table S3) and signal void size (Fig. S6). An increase in torque improves driveability of the CoSMA and allows for higher curvature bending.

S4 MR System as Catheter Sensor

The volume of metal in the MRI bore, as well as image settings, have a significant impact on the information that can be gathered from the image. A further consideration here is the time taken to harvest and post-process the images. For any practical control application this speed consideration would need further refinement (e.g. as in [17]). To prove our concept on a preclinical MR system (Bruker BioSpin, Ettlingen, Germany), comprising of a horizontal 7T magnet, a gradient system with a maximum amplitude of 660mT/m, a quadrature-driven transmit-receive volume radiofrequency coil (inner diameter 72 mm), we employed a 3D ultra-short echo time (UTE) sequence with the following parameters: echo time TE = 0.011ms; repetition time TR = 20ms; 51360 projections; field of view 100 × 60 × 60 mm, coronal orientation, reconstructed to 128 × 128 × 128 points, image resolution: 781 × 469 × 469 µm. Total acquisition time: 1027 seconds (≈17 minutes). Notably, the imaging time can be reduced by orders of magnitude by scanning a 2D plane determined by the output of any previous time step. A single 2D slice taken on the same settings generates an image in 8 seconds. Fig. S6 shows the artifacts in an example slice of the 3D UTE dataset, induced by 4 mm diameter rings of 1-4 loops (as in Supplementary Method S3.3). These can be binary thresholded into either "signal" or "void" and thus characterized as total area of void and maximum length of void (Feret diameter) with results shown in Table S4. From this, and Fig. S6, it is clear (as expected) that reduced metal volume improves image fidelity although a diminishing return can be observed - each step reduction in metal volume affords less proportional improvement in image. This diminishing relationship is highlighted in the final column - void area per unit metal volume. The increased signal void could, in future works, be mitigated by (a) reducing the overall diameter of the CoSMA, (b) via targeted signal processing or (c) by exploiting machine learning methods [17] but these investigations are beyond the scope of this publication.

The magnitude of the magnetic torque, subject to the background field exceeding the saturation

Table S3: Summary of demagnetization factors obtained via the FEM described in Supplementary Method S3.1 for the geometry shown in Fig. S4. The wire is annealed iron (99.5% purity, $m_s = 1.43 \times 10^6 A/m$) with a diameter of 0.25 mm. In the upper calculation the loop has an outer diameter of 4.0 mm and in the lower calculation an outer diameter of 2.1 mm. The magnetic torque (τ_{mag}) is calculated according to equation 1 at peak ($\theta = 45°$). Note the diminishing returns in torque as loops are increased as axial demagnetization factor reduces.

**Ring Outer Diameter = 4 mm**				
number of loops (n)	n_a	n_r	volume (mm^3)	$\tau_{mag}(mNm)$ at $\theta = 45°$
1	0.95	0.03	0.62	0.73
2	0.90	0.05	1.23	1.34
3	0.85	0.08	1.85	1.84
4	0.81	0.10	2.47	2.26
**Ring Outer Diameter = 2.1 mm**				
number of loops (n)	n_a	n_r	volume (mm^3)	$\tau_{mag}(mNm)$ at $\theta = 45°$
1	0.86	0.07	0.31	0.31
2	0.73	0.14	0.62	0.47
3	0.63	0.18	0.93	0.53
4	0.56	0.22	1.23	0.54

Table S4: Summary of metal volume, signal void area and signal void Feret diameter for the 4.0mm diameter rings (shown in Fig. S6). Void area per unit metal volume is also shown.

Number of loops (n)	Metal volume (mm^3)	Void area (mm^2)	Void Feret diameter (mm)	Void area per unit metal volume (mm^{-1})
1	0.6	370.6	27.2	601.4
2	1.2	433.6	30.0	351.8
3	1.8	519.3	32.9	280.5
4	2.5	694.6	34.1	281.7

magnetization, is invariant with increase in field. The effect of background field on artifact area was analyzed in detail in [18]. A mean equivalence is presented as:

$$\frac{A_{B_{01}}}{A_{B_{02}}} = \sqrt{\frac{B_{01}}{B_{02}}}, \tag{S5}$$

for B_0 in the range 1.5T to 7T where $A_{B_{0i}}$ is the area of the signal void associated with B_{0i}. It is evident from Fig. S6 E that decreasing B_0 from 7T will improve the image (reduce artefacts) without loss of torque and therefore manipulability. This is true down to 1.8T where the relationship becomes more complex and unpredictable but it is reasonable to assert that overall performance in a 1.5T clinical scanner would be improved over the 7T scanner. Due to the low volume and characteristic length of our ferrous components, heating effects are most likely negligible but this is something that will require further investigation when we move to a clinical scanner.

S5 Simulated Aortic Arch Navigation

We leveraged the rigid link model to ascertain the requisite base configurations for navigation into each of the five branches of the aortic arch phantom. Path centre-lines were extracted in 3D Slicer 5.8.1 (https://www.slicer.org/) and loaded into our custom interactive simulation environment, where direct operator control could explore this synthetic search-space. Using this environment,

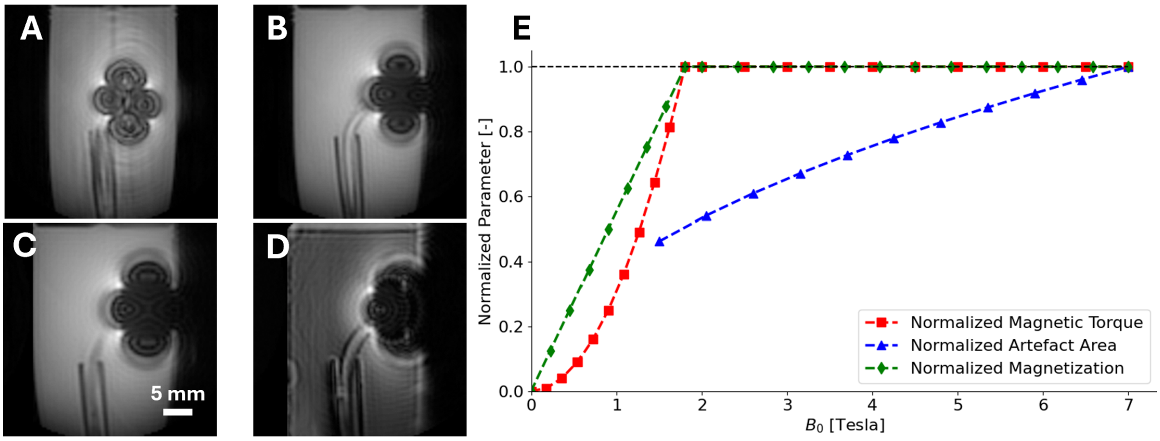

Figure S6: (**A-D**) Signal voids generated by the presence of iron rings in MR images. A 20 mm length of 3.0 mm diameter braid was affixed with 4.0 mm diameter iron rings of 1, 2, 3 and 4 loops (corresponding to Figure S4 and Tables S3 and S4. Both the size and the intensity of image artifacts/signal voids are correlated with metal volume. (**E**) Background field (B_0) against magnetic torque [13] (red), artefact area [18] (blue) and magnetization [13] (green). All variables normalized against results in the 7T scanner used in this work.

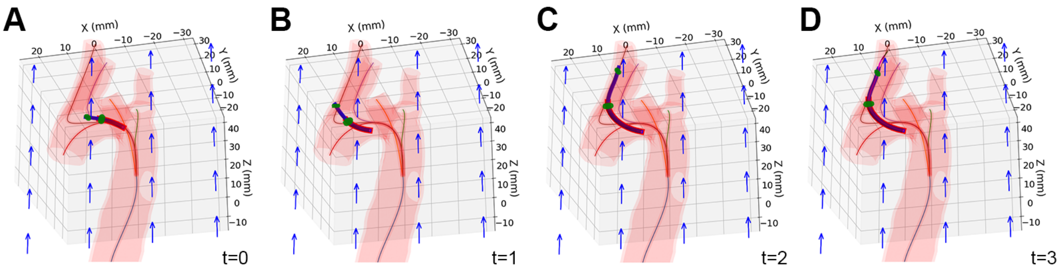

Figure S7: The four time-step simulated navigation into the right subclavian artery. The blue arrows indicate the unchanging background field B_0. Anatomical geometry outline is shown in transparent pink and centre-lines in red. The catheter follows the desired path according to the base configurations presented in Table S5. For this three-dimensional and sinuous navigation, spatial error from the geometric centre-line peaks at 4.05 mm - approximately equal to the mean radius of the anatomy through which it travels.

the base configurations to navigate each pathway in a minimal contact manner were predetermined. A more robust path-planning process is clearly a prerequisite for any control application, a task which lies beyond the scope of this work. Each of the five navigations was discretized into four equal time-steps. Base configurations and RMS error values for the four time-steps and five paths are shown in Table S5, and the results of these simulated navigations are shown in the Supplementary Video S3. Additionally, Fig. S8 shows our four time-step simulated navigation into the most sinuous of the five target arteries - the Right Subclavian Artery (RSA). As can be seen in Table S5, the spatial error is generally of the order of 1 mm and, with the exception of the RSA, below 2 mm. For the RSA navigation spatial error peaks at 4.05 mm. Considering a mean radius of the RSA of 4 mm [19] we would expect this level of error to incur some small amount of anatomical contact which is reflected in the Supplementary Video S3. For the other four navigations, with the errors presented, no anatomical contact would be expected.

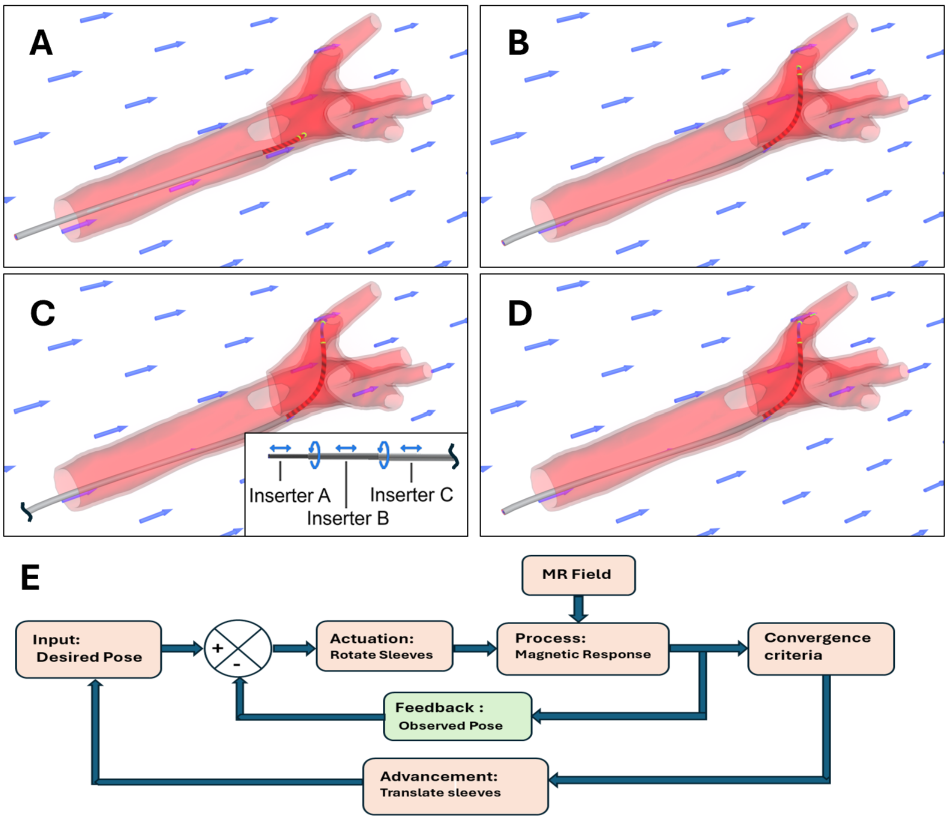

Figure S8: (A-D) Four sample insertion steps of the CoSMA as controlled by the process in (E). Desired pose derives from the preoperative scan centre-lines updated for the current state of insertion (catheter length). Initially, all sleeves are contracted together (tips coincident), translation of one or more sleeves moves to a new desired pose in accordance with Table S5. Error is closed via rotation of respective sleeves before the subsequent translation step is initiated. In the current work, the phantom is transparent and feedback comes from MR compatible cameras. In future works, after optimization of the MR sequence, feedback will be supplied from the MR Images and a controller can be implemented from this data.

Branch	Timestep	Sleeve A	Sleeve B		Sleeve C		RMS Error (mm)
		Length (mm)	Length (mm)	Roll (°)	Length (mm)	Roll (°)	
**Left Subclavian Artery (LSA)**	1	5	10	0	5	-90	1.02
	2	5	15	0	5	-90	1.62
	3	5	25	0	10	-90	1.88
	4	35	25	0	10	-90	1.29
**Left Common Carotid Artery (LCA)**	1	5	10	0	5	10	0.56
	2	5	20	0	10	10	0.95
	3	5	35	0	10	10	0.73
	4	45	35	0	10	10	1.55
**Ascending Aorta (AA)**	1	5	15	-85	10	55	1.30
	2	5	20	-85	10	60	1.02
	3	5	25	-85	15	60	0.92
	4	35	25	-85	15	60	1.18
**Right Common Carotid Artery (RCA)**	1	5	15	0	10	45	1.43
	2	30	25	0	15	45	1.67
	3	5	30	175	20	245	0.88
	4	35	30	175	20	245	1.30
**Right Subclavian Artery (RSA)**	1	5	15	0	10	85	1.80
	2	5	20	0	10	-85	2.55
	3	5	35	0	20	-80	4.05
	4	40	35	0	20	-80	4.00

Table S5: Base configurations for the navigations shown in Figures 3 and 4 determined via the simulation described in the Methods Section. The five DoFs of the catheter are shown as length of sleeve A, length and roll of sleeve B and length and roll of sleeve C. Roll refers to rotation about the local Z-axis of the sleeve in question. Error (in mm) is calculated as the RMS of the spatial error between the lumen centre-line and the simulated position of each node of the 20 rigid links (as described in Supplementary Method S1).

S6 Fabrication

With reference to Fig. S9 and Supplementary Video S4, to create sleeve A, 5 mm of 0.25 mm diameter iron wire (GF44272571 Iron Wire Reel - Merck KGaA, Darmstadt, Germany) was superglued (Everbuild HV50 Industrial Superglue - Silka Limited, Welwyn Garden City, UK) in axial alignment to a 100 mm length of 0.4 mm diameter nylon wire (Vantage Pro 1/4 Pound Transparent - Fladen Fishing AB, Varberg, Sweden). This was attached with the same superglue to an 800 mm long, 0.15 mm diameter nitinol rod (Super-Elastic Nitinol Wire - McMaster-Carr, Elmhurst, USA). For Sleeve B, the same 0.25 mm diameter iron wire was wound once orthogonally onto a 2.1 mm diameter temporary rod. The diameter of the temporary rod was determined from the 45° angle at which the iron ring must sit on the 1.5 mm diameter braided sleeve ($\sqrt{1.5^2 + 1.5^2} = 2.1$). The rod ensures the windings will be circular (as opposed to elliptical) and thus exhibit no alignment bias when exposed to the background field. This ring was superglued to a 1.5 mm diameter, 75 mm length, nylon braid (Everlasto - James Lever 1856 Ltd, Manchester, UK). For Sleeve C, a 50 mm long, 3 mm diameter braided nylon sleeve (Lead-Weight Tape 100 g - Merrick & Day, Lincolnshire, UK) was threaded onto a temporary rod of 2.5 mm diameter. The same iron wire was wound once orthogonally around this sleeve and secured with superglue.

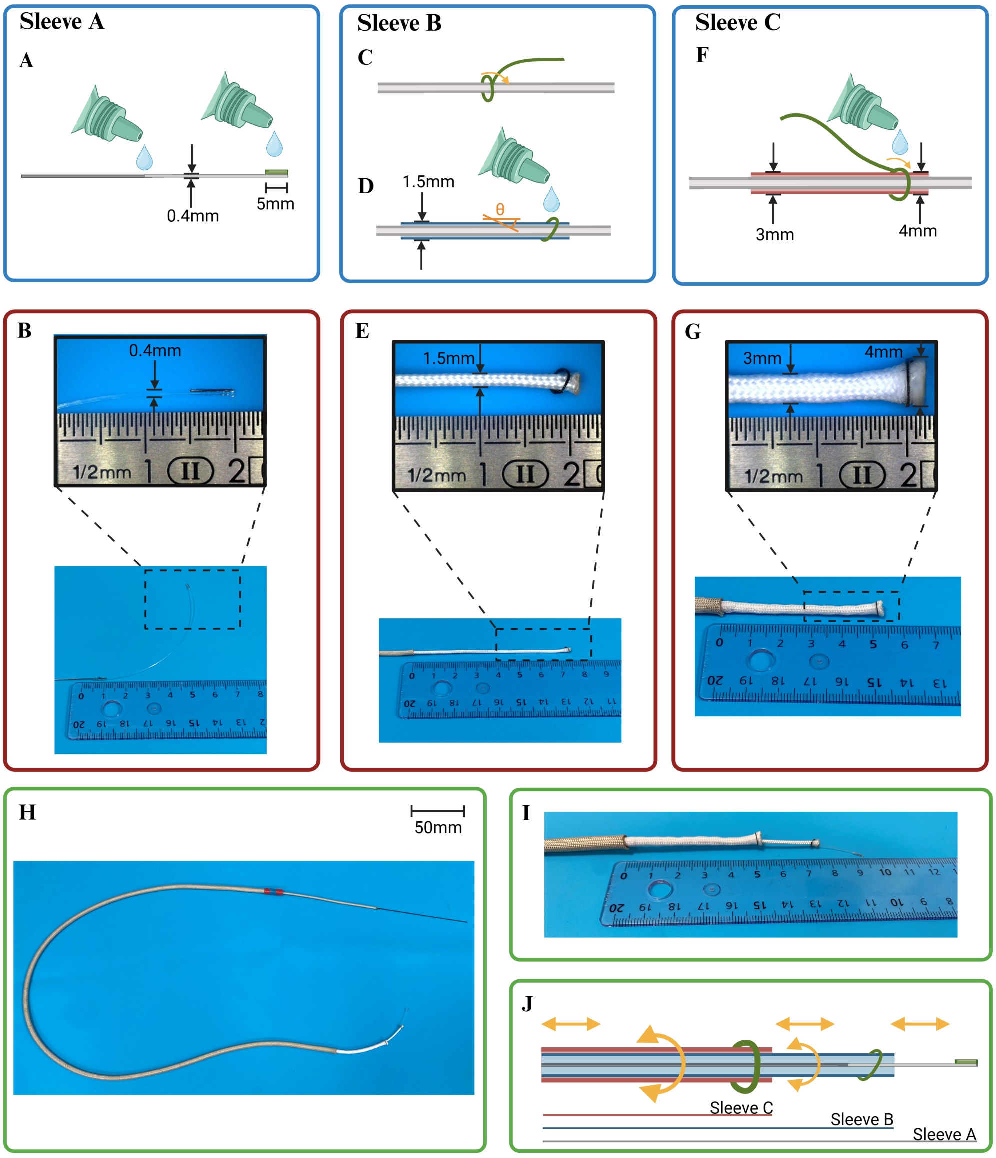

Figure S9: Fabrication Process of the CoSMA: (A,B) Sleeve A, a 5 mm iron pin is superglued to 0.4 mm diameter nylon wire. (C) Sleeve B, iron wire is wound orthogonally around a 2.1 mm diameter temporary rod. (D,E) This winding is superglued at a 45° angle onto the 1.5 mm diameter 75 mm length braid. (F,G) Sleeve C, iron wire is orthogonally wound around the 3 mm diameter, 50 mm length braid and secured with superglue. (H-J) The full shape forming catheter exhibits 5 DoFs (yellow arrows). Sleeves B and C are connected to 600 mm concentric fibreglass sleeves to enable mechanical control from outside the MRI system. (Created in BioRender. May, Y. (2026) https://BioRender.com/16hiyhn).

The base of sleeves B and C of the CoSMA was attached to $\sim$ 600 mm of concentric fibre-glass cable sleeve (Hellermann-Tyton international, www.HellermannTyton.com), via heat fusion, to allow manual mechanical control from outside of the bore of the MRI system. Finally, Sleeve A was inserted into Sleeve B and both into Sleeve C to form the full shape forming catheter shown in Fig. S9(h,i). 5 DoFs can be achieved due to independent translation of all 3 sleeves, and rotation around the central axis of Sleeves B and C (Sleeve A is axially symmetric).

Supplementary References

[1] Lloyd, Peter et al. "Optimal design of soft continuum magnetic robots under follow-the-leader shape forming actuation". In: *2020 International Symposium on Medical Robotics (ISMR).* IEEE. 2020, pp. 111–117.

[2] Abbott, Jake J, Diller, Eric, and Petruska, Andrew J. "Magnetic methods in robotics". In: *Annual Review of Control, Robotics, and Autonomous Systems* 3.1 (2020), pp. 57–90.

[3] Da Veiga, Tomás et al. "Material characterization for magnetic soft robots". In: *2021 IEEE 4th International Conference on Soft Robotics (RoboSoft).* IEEE. 2021, pp. 335–342.

[4] Rucker, Daniel Caleb and Gilbert, Hunter Bryant. *Methods for improving stability of concentric tube steerable devices using asymmetric flexural rigidity.* US Patent 11,964,387. Apr. 2024.

[5] Modes, Vincent and Burgner-Kahrs, Jessica. "Calibration of Concentric Tube Continuum Robots: Automatic Alignment of Precurved Elastic Tubes". In: *IEEE Robotics and Automation Letters* 5.1 (2020), pp. 103–110. DOI: 10.1109/LRA.2019.2946060.

[6] Qiu, Michael Y. et al. *Quantification of the flexural rigidity of endovascular surgical devices using three-point bending tests.* 2023.

[7] Nabeel, PM et al. "Measurement of Arterial Young's Elastic Modulus using ARTSENS Pen". In: *2018 IEEE International Symposium on Medical Measurements and Applications (MeMeA).* IEEE. 2018, pp. 1–6.

[8] Polio, Samuel R et al. "Cross-platform mechanical characterization of lung tissue". In: *PloS one* 13.10 (2018), e0204765.

[9] Leipzig, Nic D and Shoichet, Molly S. "The effect of substrate stiffness on adult neural stem cell behavior". In: *Biomaterials* 30.36 (2009), pp. 6867–6878.

[10] Lloyd, Peter et al. "Magnetic soft continuum robots with braided reinforcement". In: *IEEE Robotics and Automation Letters* 7.4 (2022), pp. 9770–9777.

[11] Gilbert, Hunter B, Rucker, D Caleb, and Webster III, Robert J. "Concentric tube robots: The state of the art and future directions". In: *Robotics Research: The 16th International Symposium ISRR.* Springer. 2016, pp. 253–269.

[12] Kim, Yoonho and Zhao, Xuanhe. "Magnetic soft materials and robots". In: *Chemical reviews* 122.5 (2022), pp. 5317–5364.

[13] Abbott, Jake J et al. "Modeling magnetic torque and force for controlled manipulation of soft-magnetic bodies". In: *IEEE Transactions on Robotics* 23.6 (2007), pp. 1247–1252.

[14] Beleggia, Marco, Vokoun, D, and De Graef, M. "Demagnetization factors for cylindrical shells and related shapes". In: *Journal of Magnetism and Magnetic Materials* 321.9 (2009), pp. 1306–1315.

[15] Parq, Jae-Hyeon. "Magnetometric demagnetization factors for hollow cylinders". In: *Journal of Magnetics* 22.4 (2017), pp. 550–556.

[16] Lee, Pei-Yuan et al. “Comparison of mechanical stability of elastic titanium, nickel-titanium, and stainless steel nails used in the fixation of diaphyseal long bone fractures”. In: *Materials* 11.11 (2018), p. 2159.

[17] Tiryaki, Mehmet Efe and Sitti, Metin. “Magnetic Resonance Imaging-Based Tracking and Navigation of Submillimeter-Scale Wireless Magnetic Robots”. In: *Advanced Intelligent Systems* 4.4 (2022), p. 2100178.

[18] Spronk, T, Kraff, O, Kreutner, J, et al. *Development and evaluation of a numerical simulation approach to predict metal artifacts from passive implants in MRI. Magn Reson Mater Phy.* 2021.

[19] Anantha-Narayanan, Mahesh and Nagpal, MD. “Carotid, Vertebral, and Brachiocephalic”. In: *Updates in Peripheral Vascular Intervention, An Issue of Interventional Cardiology Clinics* 9.2 (2020), p. 139.
