# Supplementary material for: A concentric tube catheter for endoluminal interventions, steered and imaged via magnetic resonance imaging
Source: Commun Eng. 2026 Mar 9;5:74. doi: 10.1038/s44172-026-00636-1 (PMC13100120; doi:10.1038/s44172-026-00636-1)
Supplement: Supplementary file 3 — Description of Additional Supplementary Files [file 44172_2026_636_MOESM3_ESM.pdf]

Description of Additional Supplementary Files:

File name: Supplementary Video S1

Description: Overview of System

File name: Supplementary Video S2

Description: Demonstration of catheter in Free Space

File name: Supplementary Video S3

Description: Demonstration of catheter in phantom

File name: Supplementary Video S4

Description: Manufacturing process of catheter
