# Supplementary figures and images for: A concentric tube catheter for endoluminal interventions, steered and imaged via magnetic resonance imaging
Source: Commun Eng. 2026 Mar 9;5:74. doi: 10.1038/s44172-026-00636-1 (PMC13100120; doi:10.1038/s44172-026-00636-1)

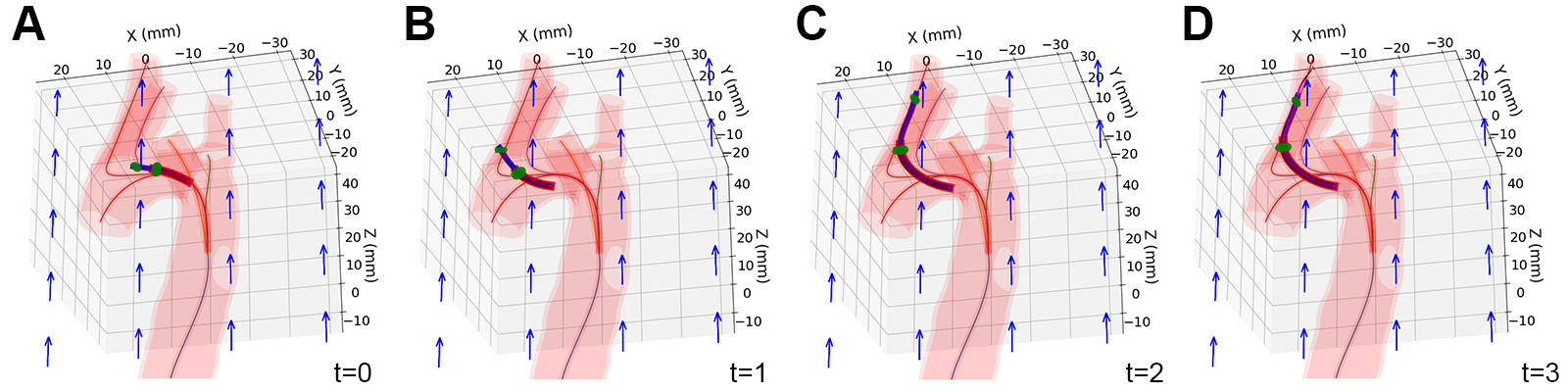

Supplement: Supplementary file 8 — Article File [file 44172_2026_636_MOESM8_ESM.zip › Images/Route5 Navigation Simulation.png]

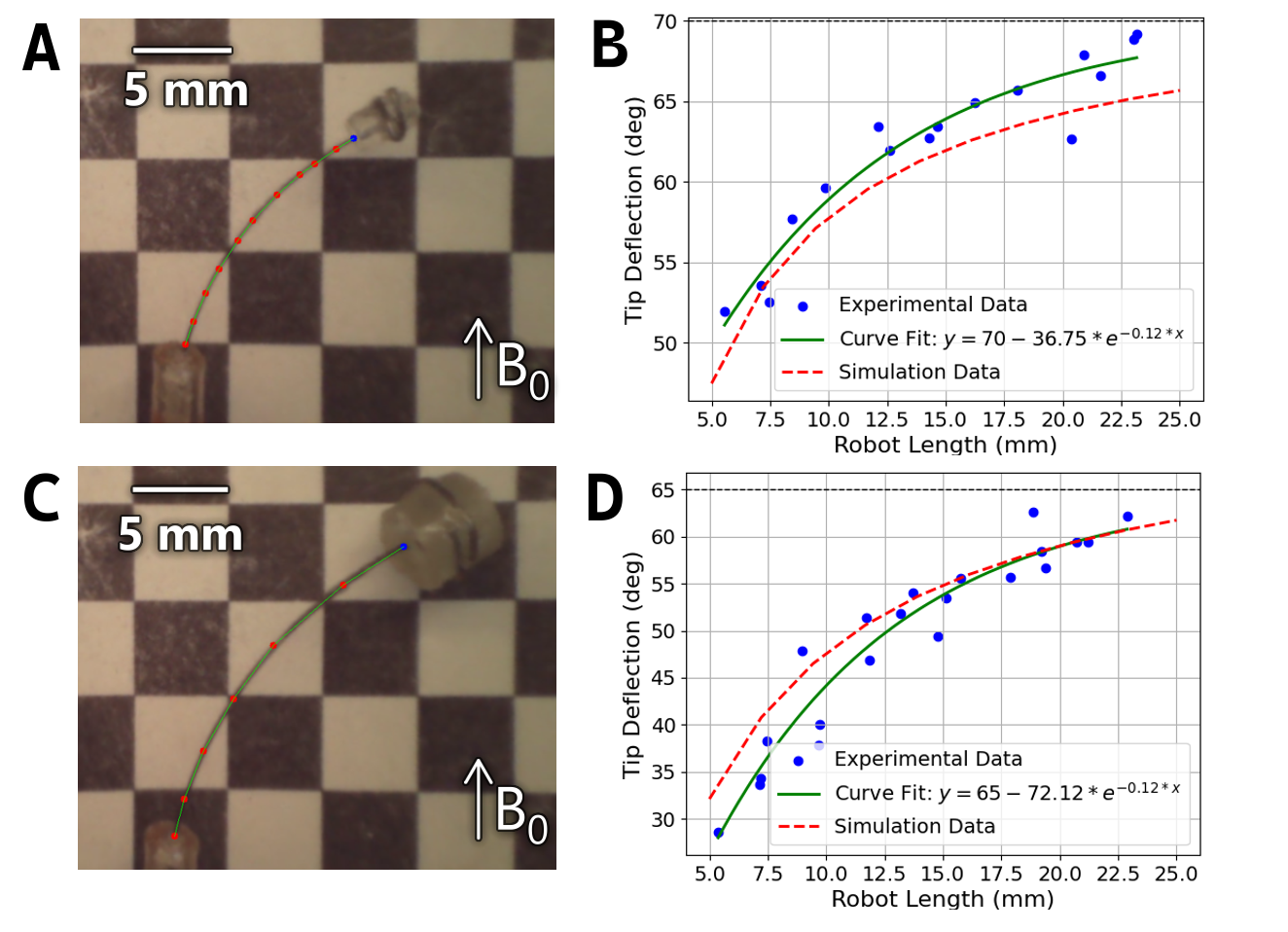

Supplement: Supplementary file 8 — Article File [file 44172_2026_636_MOESM8_ESM.zip › Images/figure_7.png]

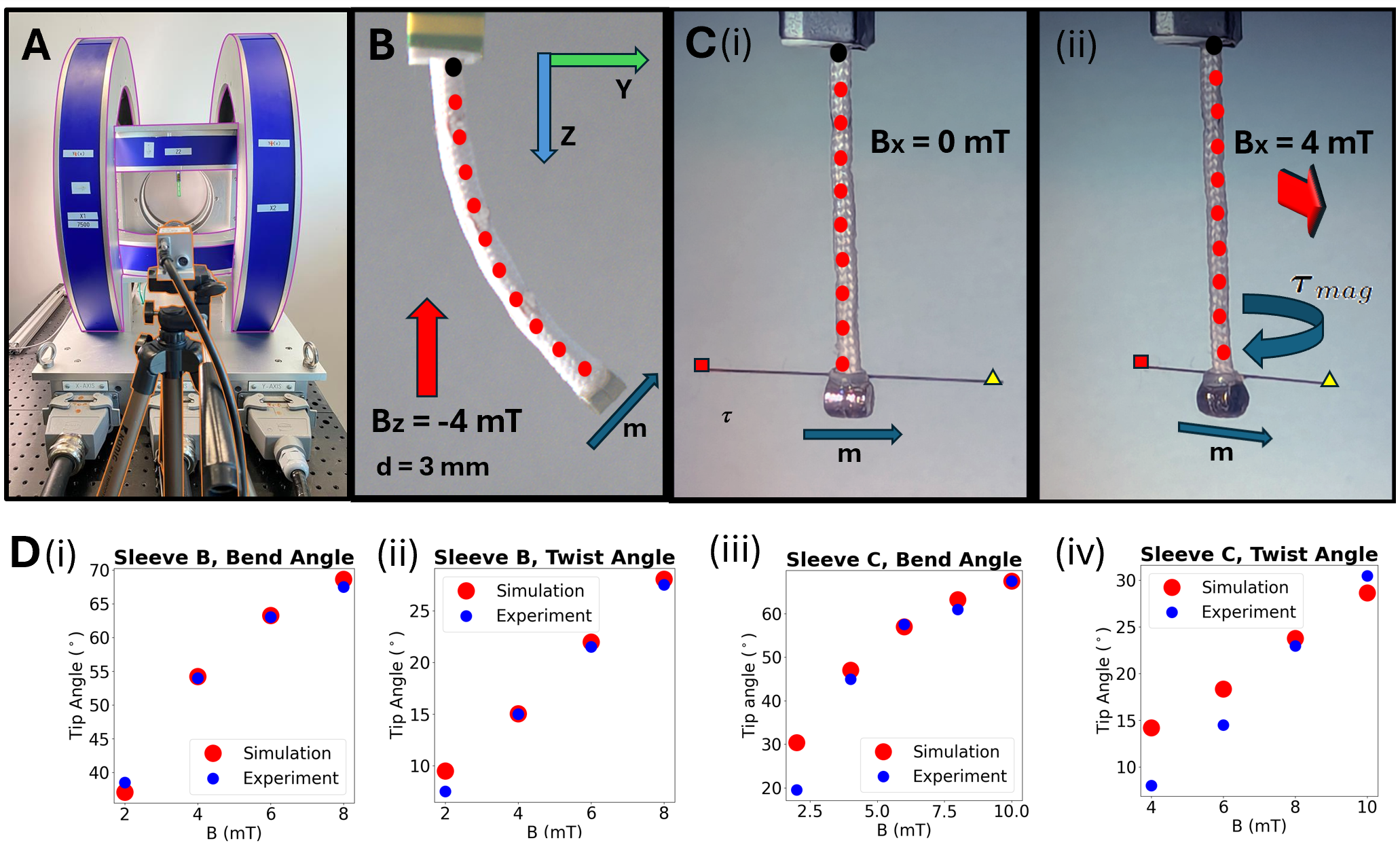

Supplement: Supplementary file 8 — Article File [file 44172_2026_636_MOESM8_ESM.zip › Images/figure_S2.png]

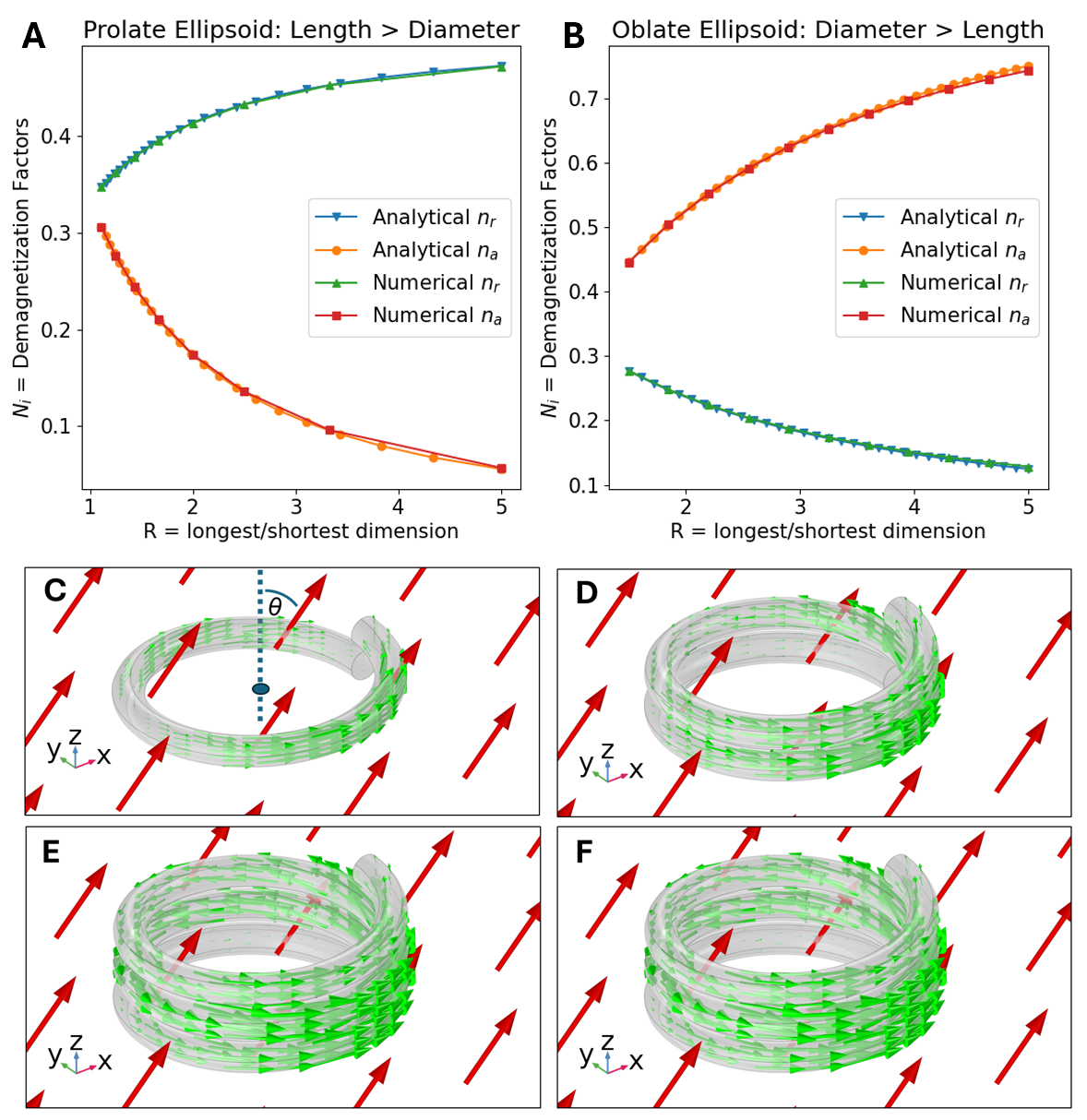

Supplement: Supplementary file 8 — Article File [file 44172_2026_636_MOESM8_ESM.zip › Images/Fig8+9.png]

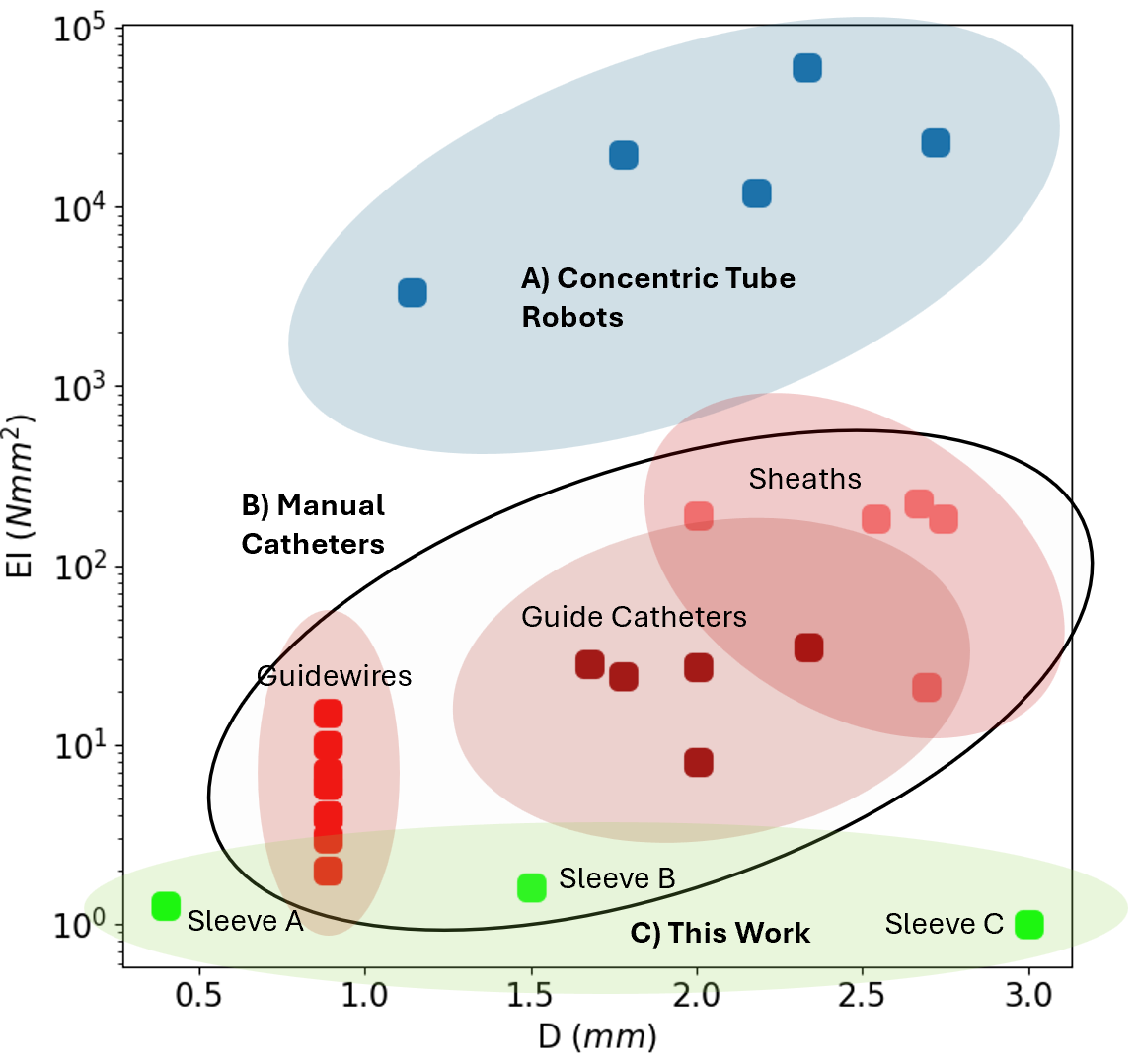

Supplement: Supplementary file 8 — Article File [file 44172_2026_636_MOESM8_ESM.zip › Images/stiffness_edited_V1.png]

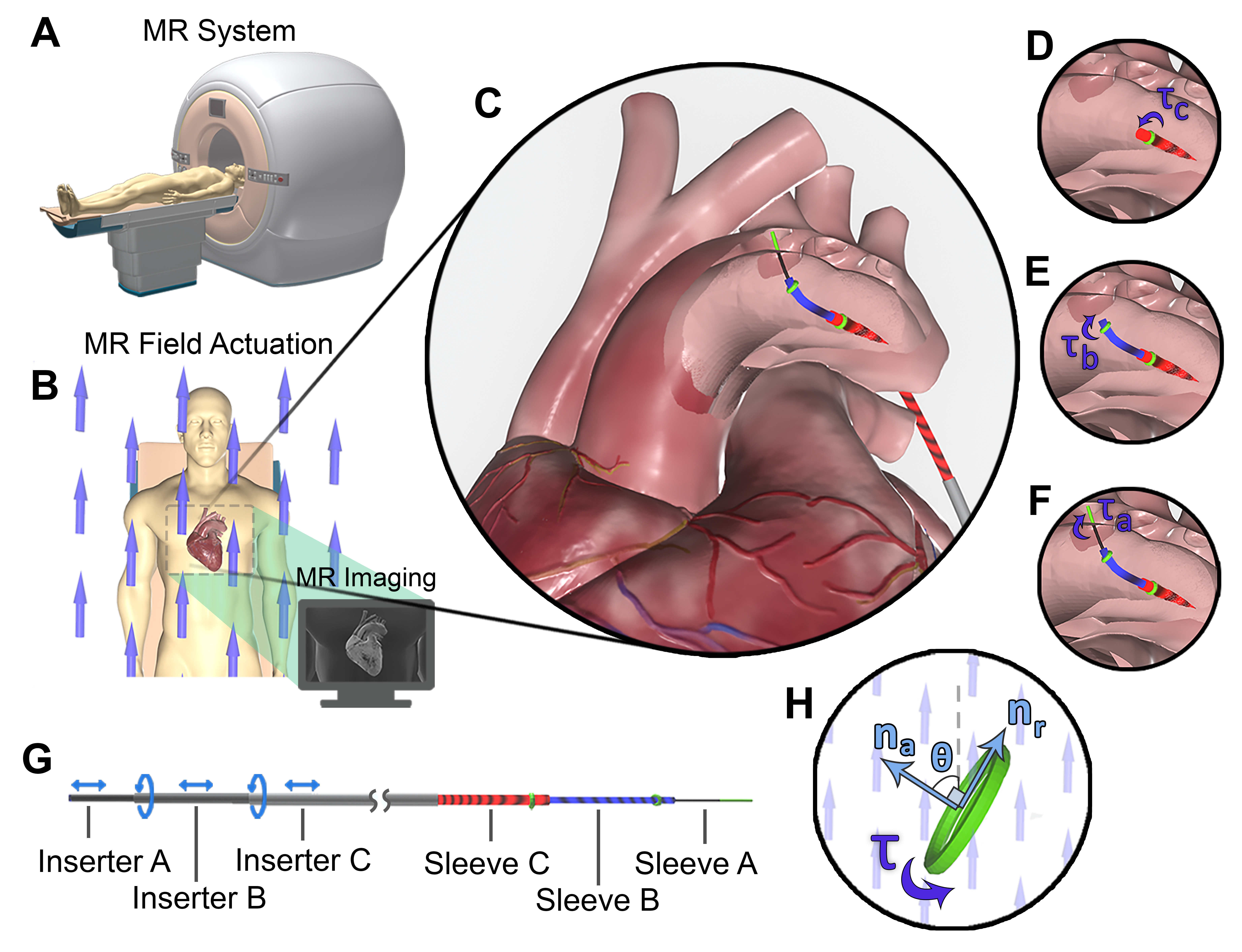

Supplement: Supplementary file 8 — Article File [file 44172_2026_636_MOESM8_ESM.zip › Images/figure_1.png]

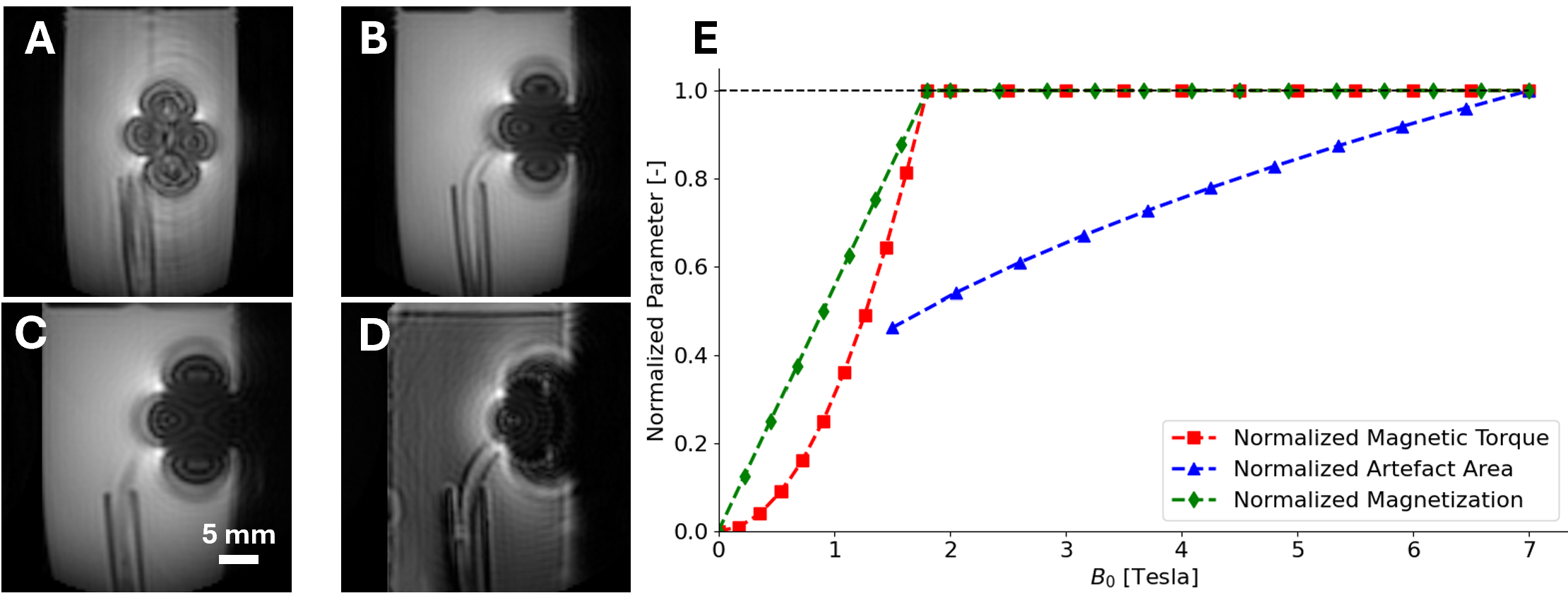

Supplement: Supplementary file 8 — Article File [file 44172_2026_636_MOESM8_ESM.zip › Images/VaryB0+Fig10.png]

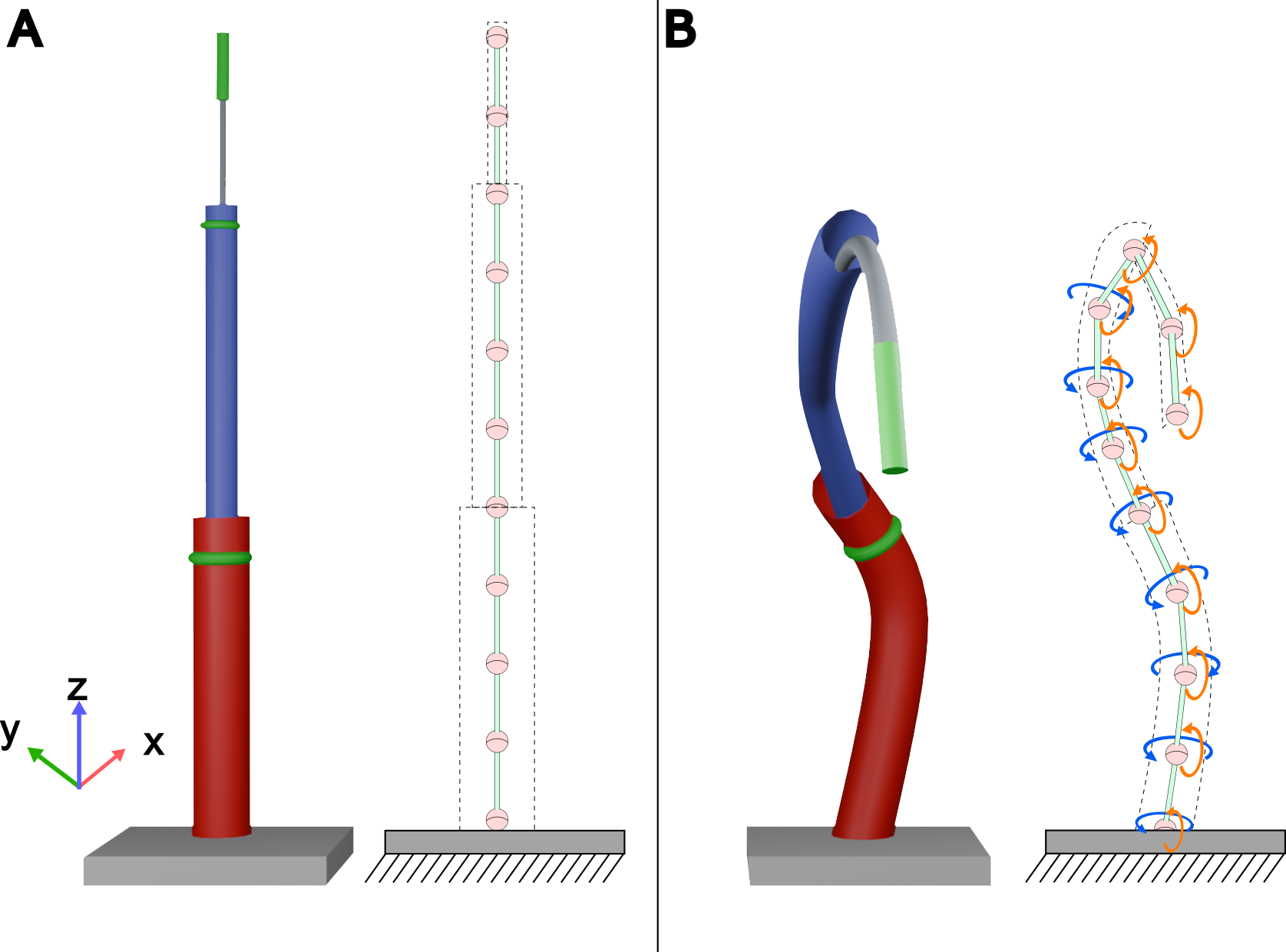

Supplement: Supplementary file 8 — Article File [file 44172_2026_636_MOESM8_ESM.zip › Images/figure_S1.png]

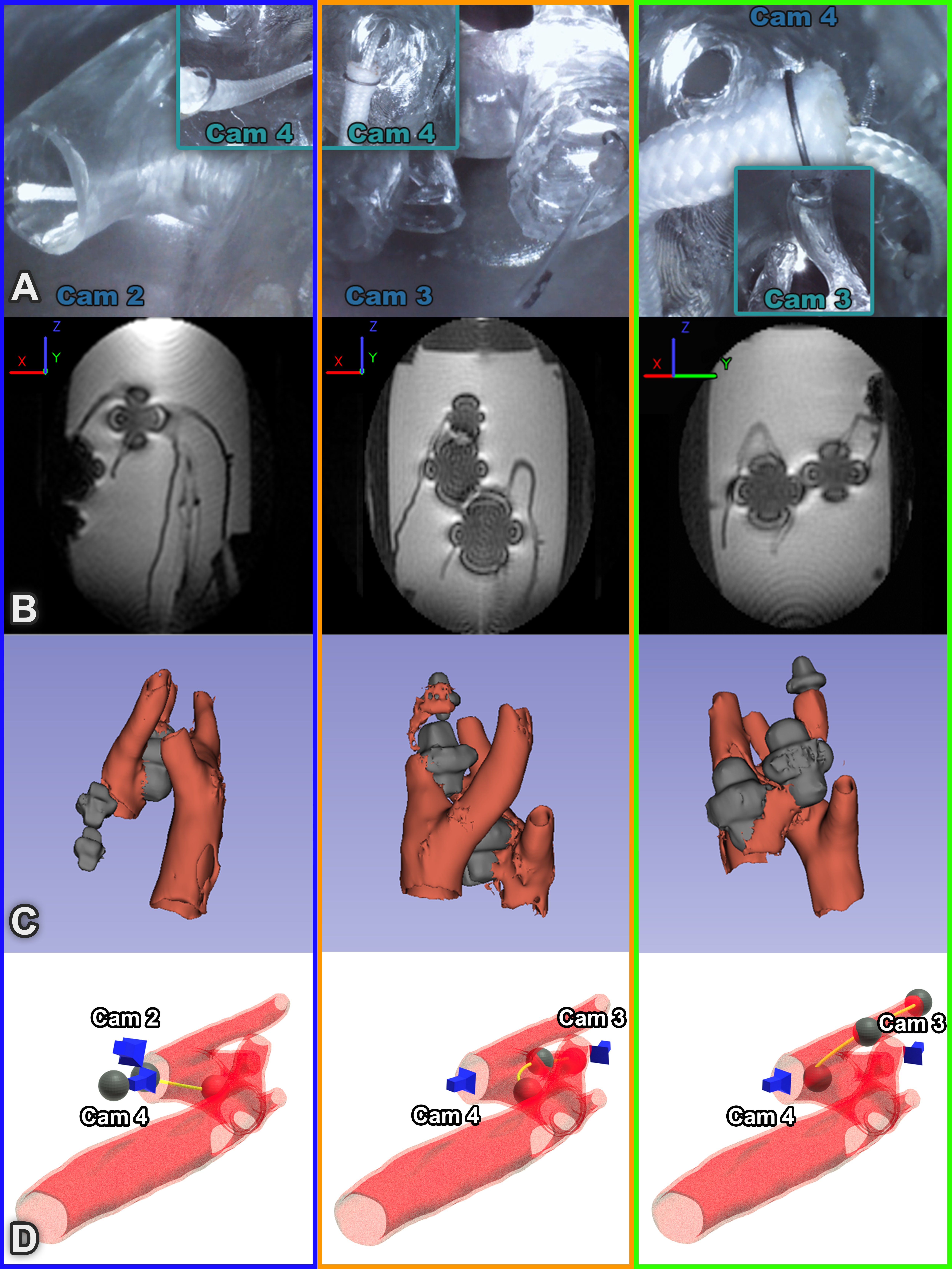

Supplement: Supplementary file 8 — Article File [file 44172_2026_636_MOESM8_ESM.zip › Images/figure_4.png]

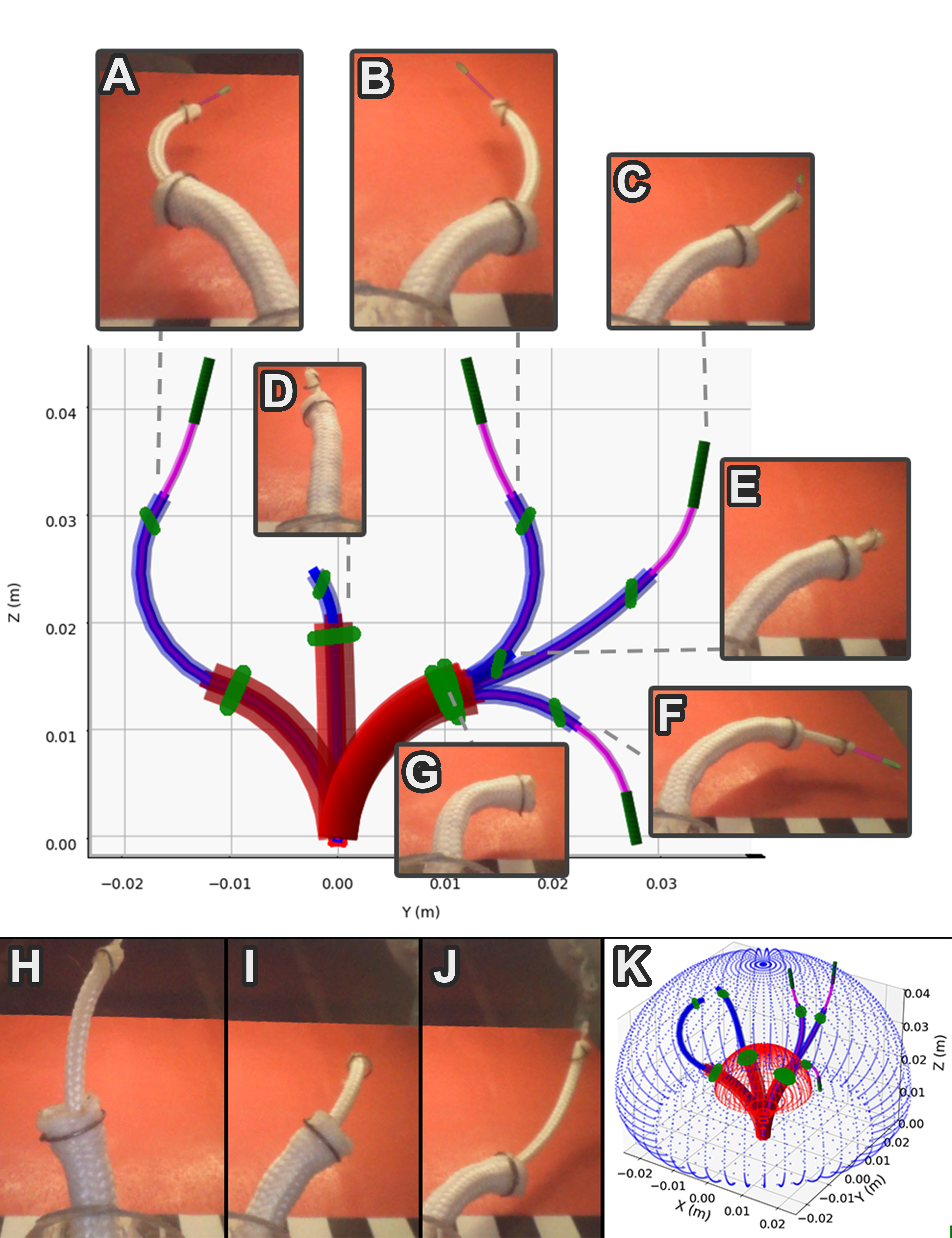

Supplement: Supplementary file 8 — Article File [file 44172_2026_636_MOESM8_ESM.zip › Images/figure_2.png]

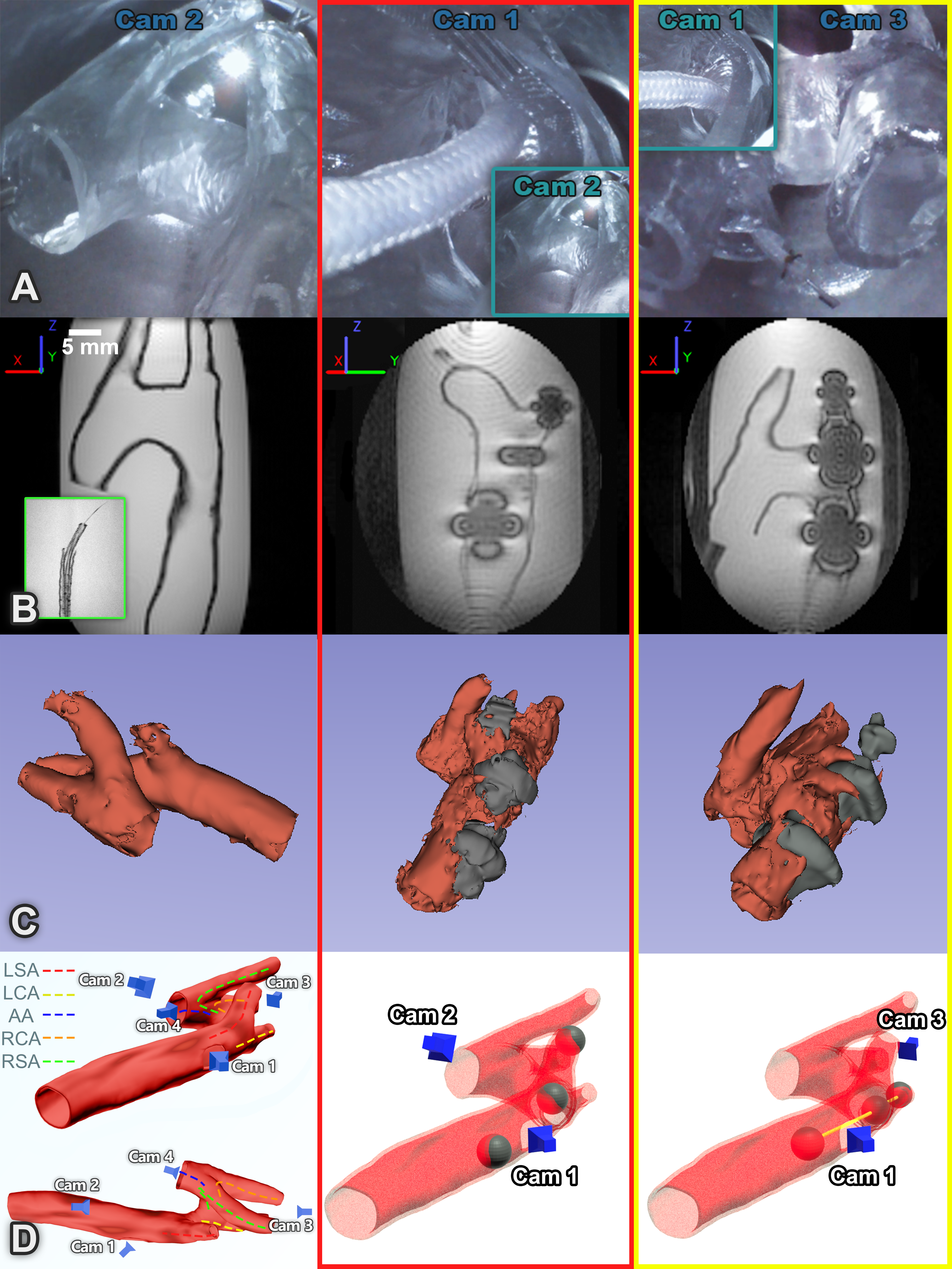

Supplement: Supplementary file 8 — Article File [file 44172_2026_636_MOESM8_ESM.zip › Images/figure_3.png]

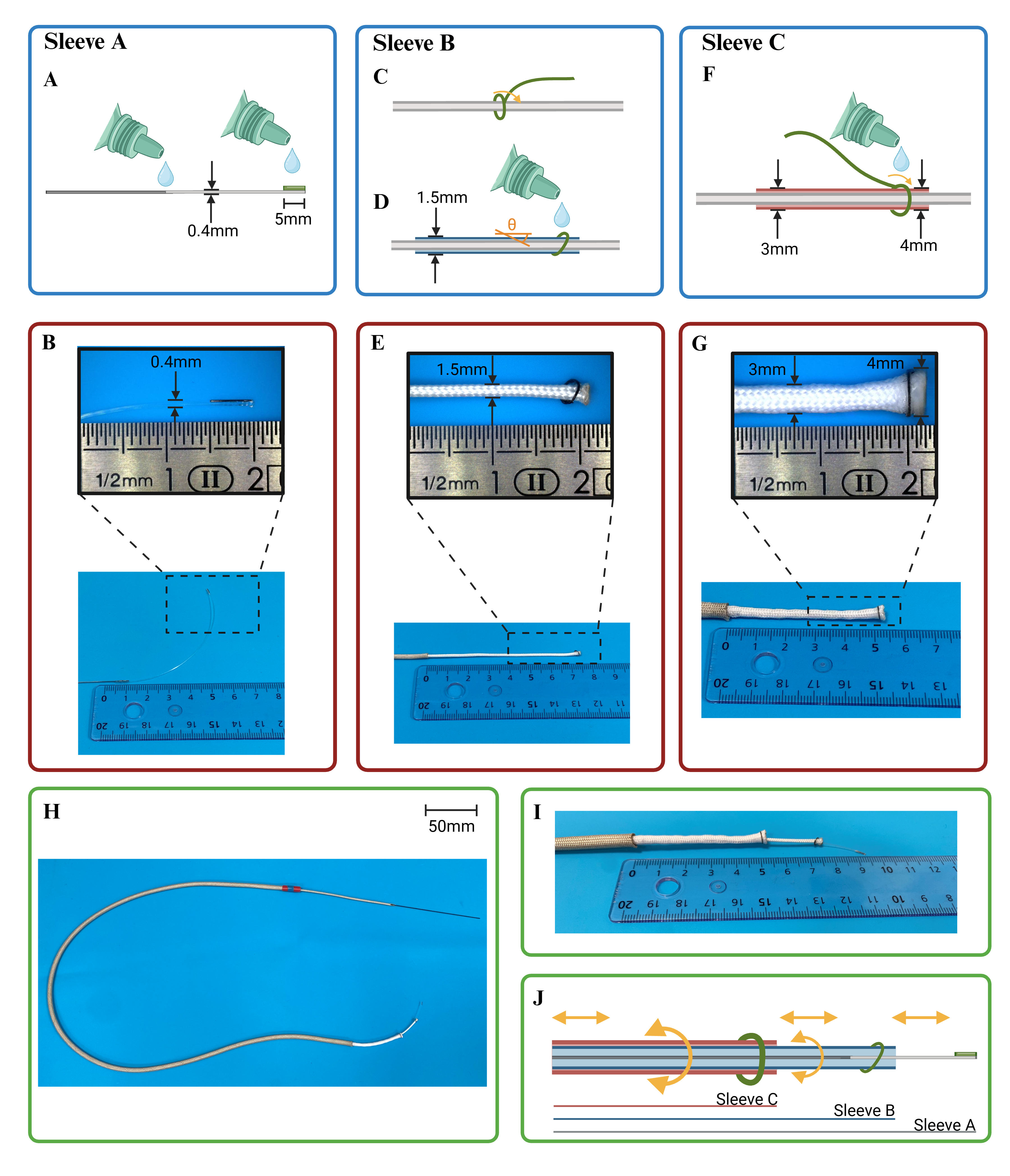

Supplement: Supplementary file 8 — Article File [file 44172_2026_636_MOESM8_ESM.zip › Images/Manufacturing_Final_License_v7.png]

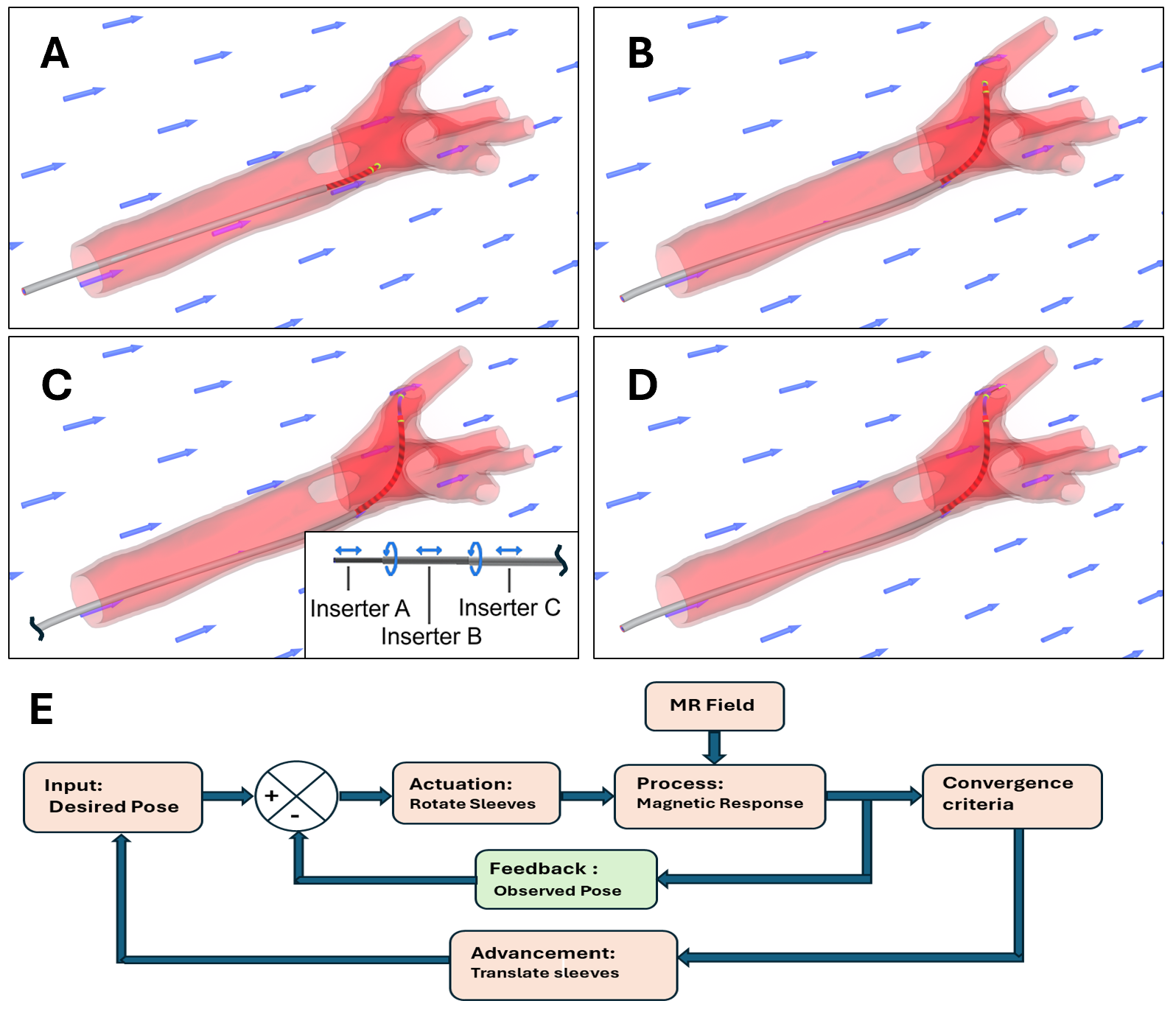

Supplement: Supplementary file 8 — Article File [file 44172_2026_636_MOESM8_ESM.zip › Images/NewBlockDiag_V1.png]
